# Dispersal in a changing world: opportunities, insights and challenges

**DOI:** 10.1186/2051-3933-1-10

**Published:** 2013-10-14

**Authors:** Sylvie VM Tesson, Pim Edelaar

**Affiliations:** Department of Biology, Lund University, Lund, Sweden; Department of Molecular Biology & Biochemical Engineering, University Pablo de Olavide, Seville, Spain; Department of Conservation Biology, Estación Biológica de Doñana – CSIC, Seville, Spain

**Keywords:** Dispersal, Global change, Opportunities, Insights, Challenges, Symposium report

## Abstract

It has been long recognised that dispersal is an important life-history trait that plays a key role in the demography and evolution of populations and species. This then suggests that dispersal play a central role in the response of populations and species to ever-increasing global change, including climate change, habitat loss and fragmentation, and biological invasions. During a symposium held at Lund University (Sweden), the causes and consequences of dispersal were discussed, and here we provide an overview of the talks. As the discussions often gravitated towards the role and our understanding of dispersal in a changing world and given the urgent challenges posed by it, we place this overview in the context of global change. We draw and discuss four conclusions: (i) methodological advances provide opportunities for improved future studies, (ii) dispersal distances can be much greater than previously thought (examples in plants and vertebrates), but also much more restricted (examples in micro-organisms), (iii) dispersal is more dynamic than we often care to admit (e.g. due to individual variation, effects of parasites, variation in life history, developmental and evolutionary responses, community impacts), (iv) using results of dispersal research for detailed prediction of outcomes under global change is currently mostly out of reach – nevertheless, that should not stop us from showing the many negative consequences of global change, and how dispersal is often a limiting factor in adapting to this.

## Introduction

Dispersal is an important life-history trait that plays a key role in the demography and evolution of populations and species (reviews [1–4]). It can be defined as the movement of individuals between natal and subsequent breeding sites, either passively or actively. The causes of dispersal remain a topic of study, but they include both ultimate factors like kin competition, inbreeding avoidance, and environmental and demographic stochasticity, and more proximate factors like variation among individuals in size, age, sex, body condition, local environmental conditions, or genetic background [1–4]. Dispersal is rarely an isolated phenomenon, since the dispersal of an organism not only has an impact on the organism itself but also on the population, community and ecosystem with which this organism interacts. Hence dispersal is a dynamic phenomenon, particularly in a changing world. Nevertheless, dispersal should play a central role in the response of populations and species to ever-increasing global changes, including climate change, habitat loss and fragmentation, and biological invasions [5]. During a symposium held at Lund University (30 January – 1 February 2013, Lund, Sweden) the causes and consequences of dispersal were discussed, pointing out and discussing the necessity to understand the role of dispersal in a changing world and the urgent challenges posed by it. Here we provide a synthesis of the talks presented at this meeting (Book of abstract: http://canmove.lu.se/sites/default/files/abstracts_final.pdf) in the context of global change. Even though the breadth of research questions, approaches and study organisms covered in the presentations was impressively wide, we first treat three emerging topics: (i) opportunities for future studies due to technical, analytical and conceptual advances, (ii) insights into the limits of dispersal, and (iii) insights with respect to the dynamical properties of dispersal, and then discuss some challenges that lie ahead.

### Opportunities: advances in dispersal methodology

Measuring dispersal and tracking dispersers is notoriously difficult, but continuous progress is being made by updating existing techniques and developing new ones, in order to better understand organism dispersal [6, 7], Tesson et al., in prep]. For instance, Clark Rushing (*University of Maryland, USA*) showed how stable isotope ratios could identify the geographic natal origin of individuals. This approach can be used to study dispersal over natural gradients of stable isotopes [8–10], but also over much smaller spatial scales by artificially enriching individuals or habitats with certain stable isotopes [11–14]. For smaller organisms, Ekvall et al. (*Lund University, Sweden*) showed how to use nanotechnologies to track zooplankton swimming behaviour in 3D by staining fluorescent nanoparticles (Quantum-dots [15]); a technique that can help understanding the causes of dispersal toward microhabitats and congeners [16–18] and the consequences of passive dispersal (e.g. water transport processes). Similarly, Jakob Löndahl (*Lund University, Sweden*) suggested combining modern monitoring techniques in aerosol samples (see [19]) to investigate microbial dispersal in different microhabitats [20, 21]. Devices are becoming more efficient, smaller in size and applicable to a greater number of organisms [7] to track individuals (including small sized, rare, out of reach or endangered) and assess if, when and why dispersal occurs.

An alternative to direct tracking is to assess *post hoc* if and when dispersal occurred. A promising new genetic technique is the analysis of environmental DNA (eDNA, reviewed in [22]). Alice Valentini (*SPYGEN, France*) showed how eDNA can detect the presence of transient dispersers or low density biological invaders in environmental (marine [23], freshwater [24], soil [25]) and blood samples [26], up to a few thousand years ago. eDNA also allows the detection of the source and direction of dispersal [27, 28], considering trophic interactions [26]. Alternatively, Paul Bentzen (*Dalhousie University, Canada*) explained how to use population genetic analyses to indirectly assess dispersal by evaluating the evolutionary effects of effective dispersal rate of successful reproducers among connected populations over longer time-frames, i.e. gene flow. He showed that the approach is sensitive enough to detect the impacts of environmental, life cycle and historical factors on dispersal [29]. For instance, a low genetic dispersal rate compared to rates of individual movements suggests that selection against dispersers due to local adaptation has occurred even when barriers to dispersal are not obvious. Further investigation of genetic dispersal rates for functional loci should bring greater understanding in the mechanisms of selection during dispersal.

Progress in sequencing techniques and faster and cheaper genetic analyses permit rapid and massive barcoding of populations/communities. Sylvie Tesson (*Lund University, Sweden*) showed how next generation sequencing technologies [30] and universal markers can be used to investigate the indirect effect of dispersal on community composition along environmental gradients in protists. Another application is the assessment of the biodiversity of passive dispersers transported by vectors/hosts (such as parasites or cryptic dispersers during vector migration [31]).

To assess organismal dispersal in the context of changing environments, Rachael Dudaniec (*University of Queensland, Australia*) proposed to use an individual-based landscape genetics approach in multi-species studies to deduce linear and non-linear relationships between genetic distance and landscape variables [32]. Staffan Bensch (*Lund University, Sweden*) explained how the compilation of a global database on avian malaria parasites can help to assess the impact of dispersal due to natural range expansion and biological invasion, which here sets the scene for novel and evolutionarily untested host-parasite-vector interactions that can result in high mortality and global extinction due to a lack of protective immunity in a host [33, 34].

### Insights: “Everything is everywhere”?

The geographical distribution of a species is a consequence of the distribution of suitable habitats and the ability of individuals to reach those. The success with which invasive species can establish and spread in non-native habitat after they have been moved there by humans is a testimony that their distributions were previously constrained by insufficient dispersal. On the other hand, it has been postulated that “Everything is everywhere”, especially when given enough time. While this clearly isn’t true for all species and all spatio-temporal scales, some data confirm that dispersal distances and rates can be much greater and more directional than we previously thought (Tesson et al., and Caplat, Birkhofer et al., in prep). For example, invasive bullfrogs already showed a wide distribution using an eDNA approach when dedicated censuses erroneously suggested they had not yet dispersed into new territory [35]. Andy Green (*Estación Biológica de Doñana, Spain*) also showed that aquatic birds can be major vectors for long-distance dispersal of ingested, but not digested, plant seeds that are normally assumed to have very different dispersal modes over shorter distances. Such long-distance dispersal by aquatic birds seems to have affected distribution ranges and population genetic structure, and may play an important and often overlooked role in the response to global change and in the spread of invasive species.

The capacity of micro-organisms for assisted or airborne dispersal in a dormant stage might suggest that geographic distributions are not constrained by dispersal. However, Wim Vyverman (*Gent University, Belgium*) showed how while indeed some species exhibit a global distribution pattern with no apparent dispersal limitation [36], other cosmopolitan species are actually composed of spatially-structured molecular lineages [37]. Progress in microscopy and genetic technologies are providing finer taxonomic resolution into e.g. cryptic and morpho-species [38–41], niche separation [42], and rare biota [43] and the results continue to add to the discussion. Moreover, Beth Okamura (*Natural History Museum, United Kingdom*) discussed the importance of local selection pressures and showed how small aquatic animals (cladocerans, rotifers and bryozoans), aquatic macrophytes, and certain micro-organisms often appear to have high dispersal rates but pronounced population genetic differentiation. The Monopolisation Hypothesis [44] proposes that this is due to founder effects during colonization followed by rapid local adaptation and increased competition which reduces the establishment of later arriving immigrants, effectively reducing dispersal among populations ([44], fish: [45]).

### Insights: dispersal is (even more) dynamic

It is well-known that dispersal rates and distances can vary a lot among individuals, e.g. due to random chance or differences in quality or investment in dispersal traits [1]. Our knowledge and appreciation of sources of individual variation are continuously expanding, and the same is true for its importance [46], including in the context of global change and barriers to dispersal (Caplat et al., in prep). For instance, morphological traits (size, shape, ability to attract water (hygroscopicity), appendices and density) alter the deposition rate in airborne micro-organisms (Jakob Löndahl) and the sedimentation of micro-organisms and spores in aquatic habitats. In a long-distant migrant songbird, habitat quality of wintering areas alters spring migration departure dates which subsequently influences natal and breeding dispersal as later-departing birds need to fly further north to find vacant habitat (Clark Rushing). Moreover, Julien Cote (*Paul Sabatier University, France*) explained how individual variation in behaviour influenced dispersal [47, 48]: individuals that are less tight to social groups and bolder disperse more than more social and shyer individuals [49], indicating that bold - asocial individuals will be the first to enter and settle in new habitat patches. This renders the patches now more attractive for shy - social individuals, facilitating the building up of a population, that then motivates the less social individuals to disperse into new habitat, etc. [48]. This may influence the dynamics of patch occupation in a metapopulation and range expansion due to climate change or biological invasion. Therefore, we should be receptive to the possibility that not only density but also the frequency of different kinds of individuals within the population can alter the dispersal characteristics of individuals (Caplat, Birkhofer et al., in prep).

Individual dispersal distances or rates may also depend heavily on the mode of dispersal, i.e. active, passive (by winds and currents) or assisted (by animal vectors). Beth Okamura and Andy Green argued that whether dispersal of parasites happens as free-living stages or within a host (endoparasites) can have marked effects, e.g. if hosts disperse greater distances, if the parasites impede or facilitate the dispersal of their hosts (e.g. [16], Tesson et al., in prep), or if parasites have complex life cycles, giving multiple opportunities for dispersal.

Genetic variation in dispersal traits should not be ignored, and dispersal traits might evolve in one or a few generations. This would make dispersal even more dynamic, and could cause issues with extrapolation in time. Nonetheless, Jörgen Ripa (*Lund University, Sweden*) suggested that surprisingly general model results exist to predict under what circumstances dispersal will be selected for, taking into account e.g. the costs of dispersal, the amount of environmental variation, and the amount of spatial autocorrelation (Ripa, in prep.).

As individuals disperse, their probability of establishment depends on the community where they arrive. Wim van der Putten (*Netherlands Institute of Ecology, The Netherlands*) explained that in response to global change and as an effect of shifting geographic ranges, current communities may become disrupted and new ones may form. He showed how range-shifting plants are exposed to less negative plant-soil feedback in their new ranges than related resident natives, while they were also better defended against above-ground herbivores [50].

## Discussion

### Dispersal research and management under global change

A major challenge is to turn results of research on dispersal into improved understanding and management of global change, and several talks touched upon this theme. Johan Ekroos (*Lund University, Sweden*) showed that the butterfly composition changed in more intensively used and more fragmented landscapes, with a loss of poorly dispersing species and habitat specialists [51]. Wim van der Putten implied that the consequences of the changes in geographic ranges for community organisation and evolution are not only hot topics in invasion biology, but perhaps also for naturally expanding native species. Whether or not changes in community structure have an effect on ecosystem functioning is not clear yet: Eva Lindström (*Uppsala University, Sweden*) found for bacterial communities that mass effect and habitat quality shape the dispersal success of bacteria across scales [52], and proposed to look more closely at taxonomic and phylogenetic selection during dispersal. Rachael Dudaniec suggested using landscape genetics tools to provide resistance maps (i.e. relative environmental suitabilities for dispersers), tailored to individual species, or modelled across multiple species. Per Jonsson (*University of Gothenburg, Sweden*) explained that empirical descriptions and surveys are needed to feed dispersal models to predict the efficacy of marine protected areas. Such models should not only consider the (passive) dispersal path and spatial expansion range, but also the interactions between species. Paul Caplat (*Lund University, Sweden*) showed how complex interactions between dispersal of a frugivore bird, food availability, and landscape structure can affect vegetation patterns. To account for the right mechanisms in multi-species systems requires more than ever conjunction of advanced modelling and high quality empirical data, making collaboration imperative.

### Modelling limitations

Nonetheless, incorporating knowledge and data on dispersal into predictive models under global change represents more than a formidable challenge. Playing the devil’s advocate, one must concur that species and communities are exposed to a range of abiotic and biotic interactions, with all sorts of demographic, evolutionary and plasticity dynamics and feed-backs, whose effects vary over spatial and temporal scales. All these properties have and will vary under global change so this suggests that today’s dispersal will not be tomorrow’s dispersal, which has the potential to really impact upon the dynamics of populations, species and communities. Even a very ambitious model must allow for stochasticity and other unknown variability like the arrival of invasive species. Simpler models may have it easier in that sense, and might be more general, but on the other hand may miss the essential detail and realism that is needed for reliable prediction. And ultimately, all models - whether realistic or general, statistical or mechanistic - would be asked to make predictions outside the range of data on which they were based, which is typically not advised because the model has not been validated for that range.

### The glass is half full – a refocus on what we do have

Should we therefore throw up our hands when asked what exactly will happen under different scenarios of global change? Perhaps not quite. We may not now, and in fact never, be able to say in detail what will happen in the future, but we do have enough studies and insight to at least point out the many things that have happened, could happen, and probably will happen (e.g. [53]). It was suggested that we should be more firm and offensive in letting policy makers know that we are sufficiently sure that global change typically has negative impacts in increasingly many ways, and that limiting or preventing further global change would be a wise policy even when exact predictions are lacking. As long as our studies continue to shed light on these complex issues and uncover new complexities and impacts of global change, we are on the right track.

## Conclusions

The tools and approaches with which we can measure or estimate general and effective dispersal rates, distances and directions continue to be improved or renewed. As we are getting better at detecting and tracking individuals, we are also getting better at understanding the potential for dispersal and the role that organismal/individual characteristics and the environment play in limiting dispersal and/or successful establishment. Nonetheless, these same data are also showing that at all hierarchical, spatial and temporal levels, the potential for variability in dispersal is present, and often in much greater degrees than is normally appreciated. Such sources of variation can and do have profound impacts on dispersal itself, and on the consequences of dispersal. Treating dispersal as a fixed, e.g. species-specific property will often be a strong assumption, which can result in overlooking fascinating processes or drawing erroneous conclusions. In view of this complexity and variability of the real world, turning knowledge and data on dispersal into predictions under global change represents more than a formidable challenge. This challenge can be partially tackled with better models, better data and interdisciplinary collaboration. On the other hand, an appreciation of exactly this complexity and the many ways in which things can go wrong should also be seen as a mayor past and ongoing contribution of research on dispersal.
